# 4256 Association Between Injury Intent and Emergency Department and Hospital Charges for Pediatric Firearm Injuries in the United States

**DOI:** 10.1017/cts.2020.371

**Published:** 2020-07-29

**Authors:** Diana Marie Bongiorno, Gia M. Badolato, Meleah D. Boyle, Jon S. Vernick, Joseph F. Levy, Monika K. Goyal

**Affiliations:** 1 Johns Hopkins University School of Medicine; 2 Children’s National Health System; 3 Johns Hopkins University Bloomberg School of Public Health

## Abstract

OBJECTIVES/GOALS: In 2016, more than 3,100 children died, and an estimated 17,000 children had non-fatal injuries, from firearms in the United States. In this study, we used hospital charges as a proxy for medical resource utilization, and compared differences in charges by intent of firearm injury among children. METHODS/STUDY POPULATION: In this cross-sectional study of the 2016 Nationwide Emergency Department Sample, we identified firearm injury cases among children aged 19 years or younger using ICD-10-CM external cause of morbidity codes. Injury intent was characterized as unintentional, assault, self-inflicted, undetermined, or due to legal intervention. We included patients treated and released from the emergency department (ED) or admitted alive to the hospital, and excluded those who were transferred or died in the ED. We used linear regressions with survey weighting to compare differences in mean healthcare charges by firearm injury intent, with and without adjustment for ED disposition. RESULTS/ANTICIPATED RESULTS: Among 12,469 cases in the weighted sample, mean age was 16.5 years, a majority were male (88.2%) and Medicaid-insured (57.8%), and 64% were discharged from the ED and 36% admitted. Injuries were 49.0% unintentional, 45.1% assault-related, and 1.8% self-inflicted. Compared to children with self-inflicted injuries (charges $115,224), children with assault-related injuries (charges $55,052; p<0.007) and unintentional injuries (charges $38,643; p<0.001) had lower mean charges per visit. Differences in charges were no longer significant after adjusting for ED disposition, as 85.8% of self-inflicted injuries were admitted, compared to 46.5% of assault-related and 24.3% of unintentional injuries. DISCUSSION/SIGNIFICANCE OF IMPACT: Although the majority of pediatric firearm-related injuries resulting in emergency department care are unintentional or assault-related, self-inflicted injuries result in greater per visit hospital charges, attributable to higher hospitalization rates, and likely due to more severe injuries.

